# An appeal for large scale production of antiretroviral drugs in Africa

**DOI:** 10.11604/pamj.2016.25.18.10658

**Published:** 2016-09-21

**Authors:** Nkamedjie Pete Patrick Martial, Isidore Sieleunou

**Affiliations:** 1 Institute for Research on Socio-economic Development and Communication (IRESCO), Yaoundé, Cameroon; 2 School of Public Health, University of Montreal, Montreal, Canada; 3 Research for Development International (R4DI), Yaoundé, Cameroon

**Keywords:** HIV/AIDS, PLHIV, antiretroviral drugs, Africa

## Abstract

The Acquired Immuno Deficiency Syndrome (AIDS) remains a major global public health challenge especially in Africa. The deadline set for the Millennium Development Goals (MDGs) has elapsed, meanwhile most low and middle income countries did not reach the targets. With regards to the fight against HIV / AIDS, many African countries show slow progress in implementing efficient and effective strategies to counter this pandemic. The fact that most HIV/AIDS programs in Sub-Saharan African countries are still very dependent on external funding to carry out their activities, including the supply of Antiretroviral Treatment (ART), highlights the concern of sustainability. So far, solutions that have been proposed are mainly symptomatic, claiming more budget commitment from government. Without rejecting this view, we call for the implementation of sustainable solutions to deal with the long term ART challenges. A way forward is to promote the establishment of an effective machinery for the manufacturing and large scale distribution of ART. In addition to the health gains, we argue that such an initiative would have a three-dimensional impact: (i) political, (ii) economic and (iii) social.

## Opinion

The Acquired Immuno Deficiency Syndrome (AIDS), caused by the Human Immunodeficiency Virus (HIV) remains a major global public health problem; particularly in the Sub-Saharan region. Despite the fact that incidence shows declining trends (2.1 million new HIV infections in 2013; a decline of 38% from 2001, when there were 3.4 million new infections), United Nations Aids (UNAIDS) estimates that 24.7 million people are living with HIV/AIDS in Sub-Saharan Africa; nearly 71% of global worldwide burden. Data also indicates that 1.1 million sub-Saharan Africans die from AIDS related causes. Antiretroviral Treatment (ART) coverage also appears to be low; only 39% of adults (15 years and older) living with HIV receive the drugs and 68% of pregnant women living with HIV received antiretroviral medicines for preventing mother to child transmission [1]. Besides having enough trained personnel for proper screening and adequate management of HIV/AIDS patients, sustainable availability of Antiretroviral ART is paramount [2]. In spite the fact that Africa bears the greatest burden of HIV/AIDS, the availability of ART in approved treatment centers ironically remains unacceptably low [1]. Government funds committed to fight HIV/AIDS is very low, over 90% of funding to HIV/AIDS programs in Africa between 2010 and 2011 were provided by international sources such as Global Fund against HIV/Aids, Tuberculosis and Malaria, World Bank, International Non-Governmental Organizations (NGOs) [3]. Of the 33 countries that constitute this region, 26 receive more than half of their funding from international sources. Nineteen of them depend on external sources for at least 75% of their expenses related to HIV/AIDS infection. Government support of HIV/AIDS programs for key populations is particularly low, international sources having contributed to over 90% of these expenditures from 2010 to 2011 [4]. In spite the fact that Africa bears the greatest burden of HIV/AIDS and thus the most ART needing, the continent ironically remains the most dependent on external ART producers and resources [1]. Such operational mechanism largely dependent on external inputs will likely result to delays in supplies and disruptions in supply chain of ART. With the current political, religious and humanitarian crises observed on the African continent today, this situation is only made worse mainly due to reallocation of funds to tackle prominent emergencies such as terrorism, malnutrition, access to water and sanitation… This suggests the need for African states to develop local pharmaceutical policies and strategies in order to minimize the extreme dependence of HIV/AIDS programs on foreign inputs. Nonetheless, solutions have been proposed to overcome this challenge, but most focus on increasing government budgets allocations to fight against HIV/AIDS, making it less likely to be realistic.

Brazil, an emerging country is a remarkable example with one of the highest ART coverage in the world [5]. The strategy developed and implemented by the Brazilian political and health authorities to achieve such an outcome should serve as a pathway and lead African countries, though the investment cost at the beginning of the implementation process may seem prohibitive. To produce such a transformational change, the country has developed its capacity to locally produce ART, with eleven drugs among the 20 currently available in the country being produced by public sector industries [6]. This strategy targeting universal coverage in ART despite demanding huge investment (investment cost) during the initiation phases, enables substantial benefits in a long run. Although some African countries like South Africa manufacture some of the ART molecules for local use, production capacity remains low and does not permit an optimum coverage of eligible persons living with HIV/AIDS (PLHIV) in the country [7]. African manufacturers depend on foreign inputs for the bulk of their production; practically all the machine, laboratory equipments and reagents, raw materials such as Active Pharmaceutical Ingredient (API), Aluminium foil for blister packaging, other labelling materials, and excipients are imported from abroad. Local contributions of production inputs in Africa countries like South Africa is limited to starches and sugars [8]. The costs of API (which are derived from raw materials) are very important because they are the largest contribution to the overall cost of ART production. Efficient API production requires substantial investment in chemical manufacturing technologies and the ready availability of raw materials and energy at competitive prices. Unfortunately, it is estimated that almost 95% of the continent’s API needs are met by imports [8, 9]. Indeed, African Traditional Medicine and the continent’s rich biodiversity represents a raw materials of choice, but over 80% of Africa’s natural raw materials have not been subjected to standard scientific evaluation. This leads to a great number of countries of the continent largely relying on India and China for imports of raw materials [10, 11]. Evidence suggests that building up local production of medicines help display greater availability and accessibility [12]. Thus, it is worth, for the African continent which harbors the majority of HIV/AIDS cases to develop its regional capacity to manufacture ART and render them accessible and affordable, at a scale that favours competition with foreign pharmaceutical firms. This will not only improve the reliability and sustainability of drug supply chain, and therefore benefits in terms of public health, but will also be a precursor of socio-economic and political benefits.

### Health gains

Throughout the progress of HIV/AIDS infection physiopathology, outcome of AIDS is usually associated with poor adherence to treatment protocol which may be caused by a rupture in ART supply chain [13]. Hence, disease prognosis will depend on the availability, accessibility of ART and adherence of HIV/AIDS eligible patients to the therapeutic protocol. In set of opportunistic infections due to improper treatment requires additional medication, which implies extra financial burden for patients and their families, accentuating poverty and perpetuating the cycle “poverty - disease”. So putting in place ART manufacturing plants, defining good drug distribution networks and strategies, coupled to close monitoring and follow-up of patient to ensure compliance to treatment will lead to direct significant reductions in morbidity and co-morbidities; by a decreased incidence of opportunistic diseases in these patients. In addition, tuberculosis which is the major cause of death among HIV/AIDS [13] would be greatly reduced leading to considerable reduction in mortality rates.

### Political advantages of ART manufacturing plants

Politically, the manufacture of ART requires a real and strong cooperation between the countries involved for the transfer of skills, experience and technology. In a rapidly changing world, addressing health challenges, exacerbated by factors such as climate change and mutations of disease causing vectors, need effective collaboration. ART manufacturing plants represents a strategic point to create new; and strengthen existing diplomatic and political relations between different African states. Moreover, collaborating for the implementation of ART manufacturing industries on the continent may represent a start point from which collaboration can be extended to other public health challenges, a cornerstone in speeding up achievement of the ambitious universal access to quality health care and Sustainable Development Goals (SDGs).

### Social and economic benefits

From an economic perspective, local production of ART at large scale is undoubtedly associated with huge profits. The construction and maintenance of heavy industries as well as the processes involved in drug manufacturing requires extensive man power. This stands as a path way that can greatly contribute in reducing the persistent and growing problem of unemployment on the African continent. Furthermore, high expenses generally associated with customs fees and transportation costs can be greatly reduced, yielding enormous savings. The Brazilian policy for access ART implemented by the state clearly illustrates this principle as it enabled savings estimated to approximately US $ 200 millions from 1999 to 2001 [6]. Data reveals that HIV/AIDS burden is greatest for the age group 15 to 24 who are amongst the most productive sub-populations. High scale production of ART to treat HIV/AIDS patients therefore means maintaining the highly productive man power of African nations, thus fostering productivity and economic growth [14]. However, figures on financial savings are underestimates because they do not take into account other aspects like the net social benefit associated with the management of PLHIV; variables not easily quantified such as the number of children who do not end up as orphans, associated benefits as lecturers continue to reinforce capacity, improved Quality adjusted Life Years (QALYs) associated with ART, psychosocial well-being of HIV-positive patients and their families as well as social equilibrium [6]. Health and sustainable socioeconomic development are closely related entities. The vision of universal coverage in healthcare and the achievement of the novel SDGs initiative represent ambitious targets requiring considerable time, commitment and investment. To speed up the process towards meeting up with these targets, it is primordial that self-financing and self-sufficiency means be developed. This is a foundation to ensure sustainability in the availability of ART even when the mobilization of external funds becomes less effective or undergoes a significant reduction; usually accompanied with large-scale and long term adverse impacts. However, emphasis should be laid on the fact that, the implementation of local production firms by African countries relies on the perception of the stakes and challenges by various stakeholders (African states, NGOs, pharmaceutical companies, civil society organizations, Associations of PLHIV ..) backed up by a real political commitment. Close collaboration amongst the various African states, between different sectors appears to be pivotal for the success of large scale ART production. An initiative which would certainly lead Africa in making substantial contributions in achieving components number 1, 3, 8 and 9 of the SDGs.
